# Tapentadol versus tramadol in the management of low back pain in the emergency department: Impact of use on the need for reassessments

**DOI:** 10.1097/MD.0000000000008403

**Published:** 2017-11-10

**Authors:** Carlos A. Guillén-Astete, César Cardona-Carballo, Cristina de la Casa-Resino

**Affiliations:** a Rheumatology Department; b Emergency Department, University Hospital Ramón y Cajal; c Emergency Department, University Hospital La Paz, Madrid, Spain.

**Keywords:** emergency department, pain, rachialgia (back pain), tapentadol, treatment

## Abstract

Nontraumatic musculoskeletal disorders are the main reason for presentation to the emergency department (ED), with rachialgia (back pain) being the most common reason to request medical assessment among them. This also generates the highest demand for reassessments due to poor pain control or onset of adverse reactions to the treatment prescribed in the initial assessment.

A retrospective observational study based on usual clinical practice was conducted in patients attending the ED due to low back pain during a period of 24 months. The primary objective was to determine the demand for reassessments in the ED by these patients in the following 30 days, according to the type of therapeutic approach used in the initial assessment.

A total of 732 patients who requested medical assessment due to back pain in the ED of our hospital were analyzed, 91 of whom were treated with tapentadol whereas 641 received another treatment. In the first month after the initial assessment, reassessments were less common in the tapentadol group; this difference was significant from days 8 to 15 (*P =* 0.001, odds ratio [OR] 0.252 with 95% confidence interval [CI] 0.100–0.635) and days 15 to 30 (*P <* 10^−4^, OR 0.277 with 95% CI 0.136–0.563). Patients who received tapentadol also had a better clinical evolution of pain compared to those who did not receive it (*P <* 10^−4^) and to those who received tramadol (*P <* 10^−4^).

In this study in patients with back pain, tapentadol shows clear advantages over the other analgesics analyzed, in terms of pain control and less need for reassessments.

## 1 Introduction

There is no equivalent translation for *recidivism* in the Spanish language. In English-language medical and scientific literature, the term *recidivism* refers to the repeated demand for medical assessments by the same individual, due to the same complaint, or with the events derived from the management of that complaint.^[1,2]^

This demand for new medical assessments represents a major challenge for the healthcare management in hospital and nonhospital units that attend to medical emergencies, and consequently for their efficient indexes. This is especially remarkable in Spain, where emergency departments (EDs) tend to be overused by the population.^[3,4]^

Recent observational studies have shown that the most frequently assessed complaint in EDs is nontraumatic musculoskeletal pain, reaching a prevalence of around 15%.^[5–8]^ Those conditions also generates the highest ratio of recidivism.^[5]^ These studies have also shown that, within nontraumatic musculoskeletal disorders, axial lumbar pain (low back pain) stands out as the most common chief complaint in EDs in all the age groups evaluated.^[9–11]^ Together, this leads to an increase in the care burden and healthcare expenditure, in addition to the social cost derived from the incapacity for work that it generates.^[5]^ In this context, there is a need to study the possible factors that may be causing this demand for reassessments by patients who attend EDs for back pain, to establish actions that can improve clinical outcomes in these patients after the initial assessment, reduce recidivism, and increase the efficiency of the healthcare system as a whole.

Tapentadol is a centrally acting opioid analgesic that acts as a mu-receptor agonist and norepinephrine reuptake inhibitor. It is indicated for the management of severe chronic pain that can only be adequately treated with an opioid analgesic.^[12]^ The Spanish Medicines Agency approved its use in 2010,^[12]^ and since then it has been incorporated into the management of chronic pain in EDs.

This study was proposed with the aim of analyzing the outcomes obtained by patients with low back pain treated in our ED, in terms of the need for reassessments as well as other secondary characteristics such as pain control, time off work and future need for specialized assessments. The type of drug treatment prescribed in the ED was used as a classification criterion, differentiating between those who received tapentadol and those who did not for the analysis.

## 2 Methods

A retrospective observational study was conducted in patients who attended our ED for back pain during a period of 24 months. The primary objective or endpoint of the study was to determine revisits to the ED for back pain (REB) in patients seen in our ED, according to the type of therapeutic management used.

The study included patients of both sexes from our hospital's influence area who had been diagnosed with any type of back pain in our ED from January 2014 to December 2015 (24 months). Patients in whom signs of inflammatory back pain (spondyloarthritis of any nature) were identified were excluded, as were those in whom radiological data were missing from the patient studies performed or these studies could not be assessed, and those who presented incomplete demographic data in the ED or primary care records. Patients treated with buprenorphine, fentanile, oxycodone, or oxicodone/naloxone were excluded due to its relative insignificant number (<5 cases for each treatment).

We retrospectively reviewed the medical records of these patients from the first assessment in the ED and for the following 3 months. The information was obtained from the Hospital Ramón y Cajal ED database of care records (via the Excalibur software program, Madrid, Spain), the database of care records of the Primary Care Health Centres in our hospital catchment area (via the Horus interface) and the electronic medical records of the Hospital Ramón y Cajal ED (via the Cajal software program, Madrid, Spain).

The variables considered were sex, age, time from onset of painful symptoms to the first visit, presence of comorbidities (heart failure, diabetes, hypertension, renal failure), concomitant treatment (oral anticoagulation, oral antidiabetic drugs, insulin, diuretics, corticosteroids), Kellgren–Lawrence radiological classification grade, intensity of pain at the start of treatment assessed according to a visual analog scale (VAS, with scoring from 0 to 10 and classified as: absence of pain 0, mild pain between 1 and 4, moderate between 5 and 7 and severe between 8 and 10) and Short Form-36 Health Survey (SF-36, with worst score 0 and best score 100), treatment administered in the ED, number of repeat visits to the ED for the same complaint or for complaints derived from the medication administered during the first month since the first visit, number of sick days the patient had, number of appointments scheduled in the 3 months following the initial assessment, patients who were not given an appointment in either specialized outpatients or in primary care in the 3 months following the assessment, and changes in pain intensity (VAS and SF-36) at 7 days.

In order to calculate the sample size, we used the hypothesis related to the primary endpoint: “H_1_: There are differences in the REB according to the type of treatment administered” versus “H_0_: There are no differences in the REB according to the type of treatment administered.” No studies have measured the REB according to the treatments prescribed, although around 16% (5.6) of all patients who consult for a musculoskeletal condition are known to revisit the ED within the first month for the same complaint. Assuming 16% as the worst-case scenario, proposing a reduction in the demand for reassessments of 5 percentage points (i.e., 11%) and for a 95% confidence level, with a statistical power of 80%, and assuming 15% incomplete records, 679 patients needed to be included.

The software used for data analysis was STATA version 12.0 (StataCorp). A descriptive analysis was performed of all the study variables (qualitative and quantitative). The association between qualitative effectiveness variables (REB) and the treatment groups was evaluated using the χ^2^ test or Fisher's exact test. The behavior of the quantitative variables (time, VAS) was analyzed by treatment group using the Student's *t* test or non-parametric tests (Mann–Whitney, Wilcoxon, Kruskal–Wallis) in the case of asymmetry. Logistic regression models were fitted, to evaluate the association of those variables with a *P*-value <0.15 in the crude analysis. Kaplan–Meier curves were constructed to evaluate the rate of events in time and Cox models were fitted. A stratified analysis was also performed to identify possible treatment effect modifications. Accordingly, all these treatment modifications were considered therapeutic failures and ended the Kaplan–Meier curve. In all the hypothesis testing, the null hypothesis was rejected with a type I error or α error <0.05. This study was approved by our institution's independent ethics committee.

### 2.1 Ethical responsibilities

This study was conducted in accordance with Good Clinical Practice Guidelines and the Declaration of Helsinki, and current applicable legislation.

### 2.2 Human subject protection

No patient was interviewed in person or by telephone, nor did they undergo any diagnostic or therapeutic tests.

### 2.3 Data confidentiality

The authors declare that they have followed their center's protocols regarding the publication of patient data.

### 2.4 Right to privacy and informed consent

The authors declare that no patient data appear in this article.

## 3 Results

A total of 732 patients diagnosed with back pain in the ED were analyses, of whom 91 received drug treatment with tapentadol (tapentadol group). The remaining 641 patients—who did not receive tapentadol—were considered the control group. Table 1 shows the baseline characteristics at the first visit to the ED of the patients analyzed in the study.

**Table 1:**
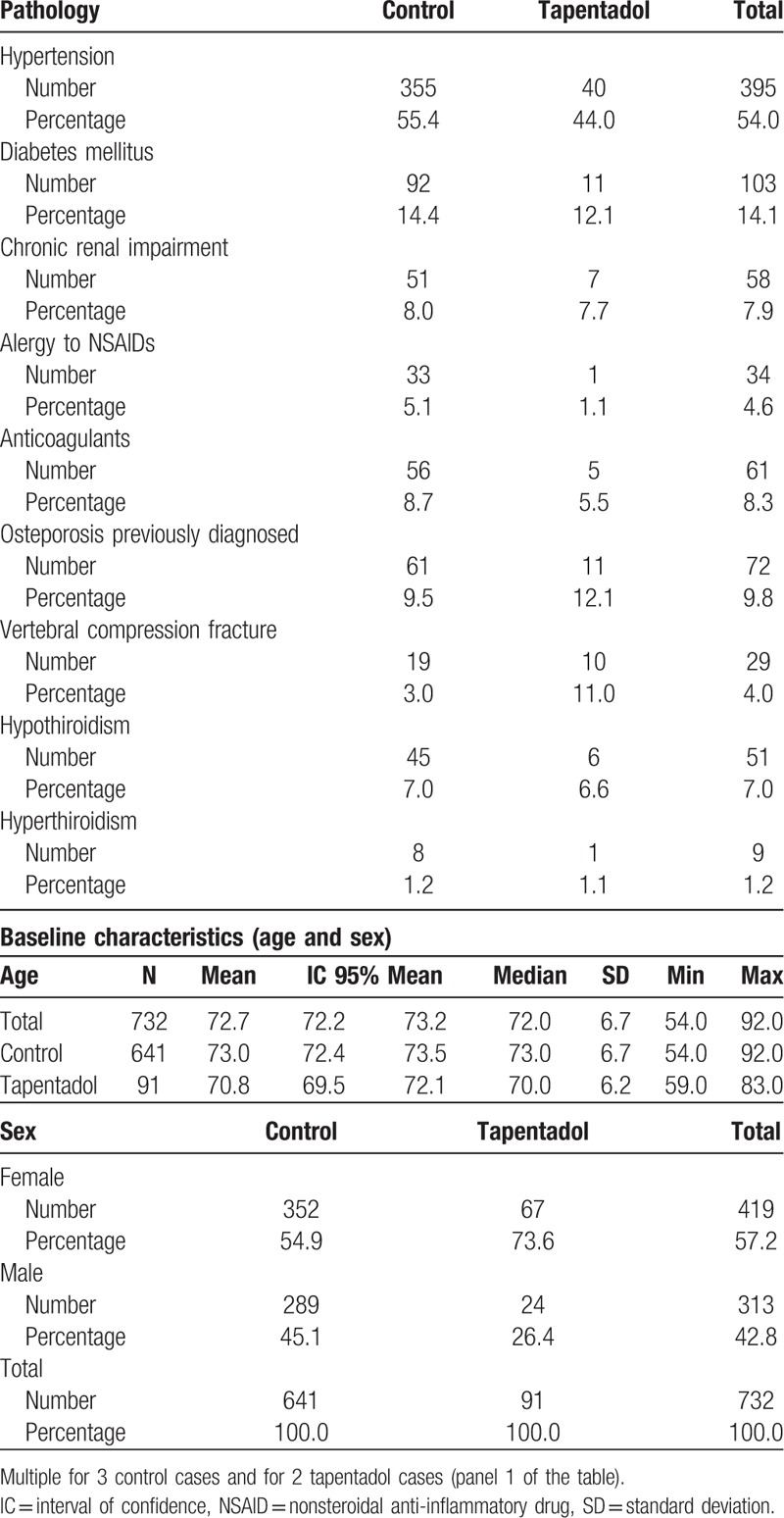
Associated comorbidities of the study population at the first visit.

Patients in the tapentadol group were significantly younger (*P *= 0.005), with the percentage of men significantly lower in the tapentadol group compared with the control group (*P* = 0.001). With respect to the associated comorbidity, hypertension, and compression fractures of the back were significantly more common in the tapentadol group (*P* = 0.044 and 0.001, respectively). The Kellgren–Lawrence radiological classification grade was significantly higher in the tapentadol group (*P* < 10^−4^); no significant association was observed between the grade and baseline VAS or short form health survey 36 (SF-36) scores. Regarding the intensity of pain, in the tapentadol group, the baseline VAS was significantly higher and the SF-36 significantly lower (*P* < 10^−4^ in both cases).

Regarding the drug treatment initially administered, of the 91 patients who were prescribed tapentadol, 23 received 25 mg twice daily and 68 received 50 mg twice daily; of the 641 patients in the control group, that is, those who did not receive tapentadol, 414 received tramadol; 44 of these received a total daily dose of ≤37.5 mg/d, 141 received a total daily dose >37.5 and ≤100 mg, 172 received a total daily dose >100 mg and ≤200 mg, and 57 received a total daily dose >200 mg.

Furthermore, some of the patients—especially in the control group and less frequently in the tapentadol group—also received a nonsteroidal anti-inflammatory drug (NSAID). Table 2 shows the frequency of the different NSAIDs (mutually exclusive) administered to patients in the control group and in the tapentadol group.

**Table 2:**
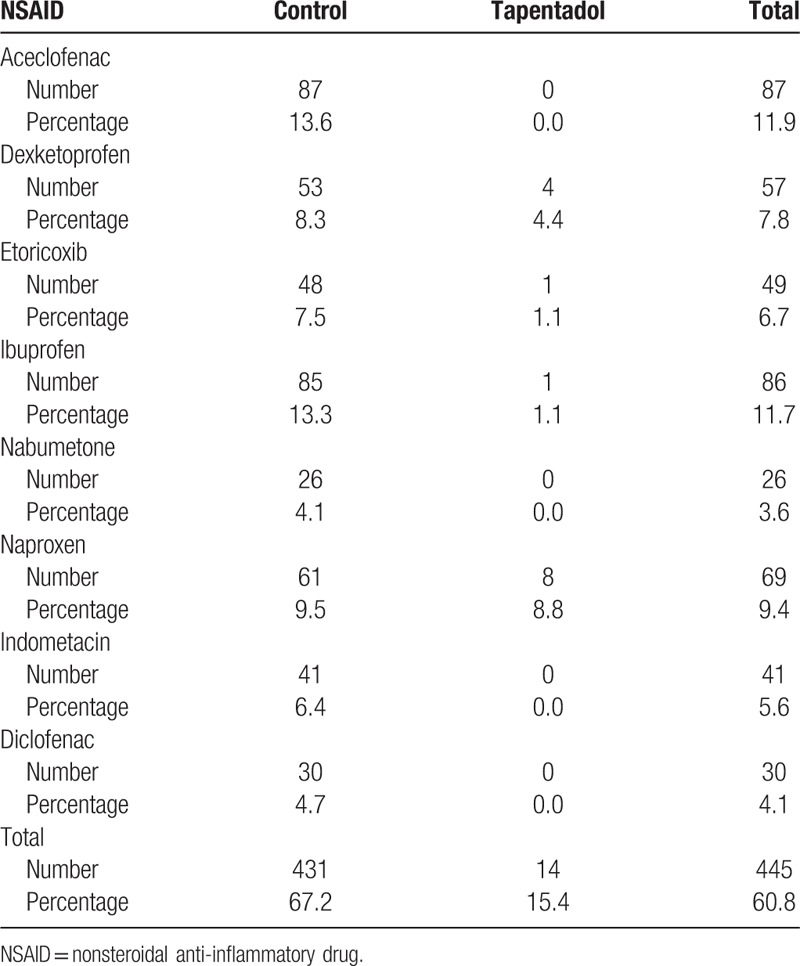
Frequency of the different nonsteroidal anti-inflammatory drugs (mutually exclusive) administered to patients in the control group and in the tapentadol group.

Patients who received tapentadol had better clinical evolution of pain (reduction in VAS and increase in SF-36) in the 7 days following the initial assessment, compared with patients in the control group (*P* < 10^−4^), although it should be taken into account that there are more exceptions due to missing baseline values in the control group. It is important to note that this better clinical evolution of pain in patients who received tapentadol was also observed with respect to patients who received tramadol (*P* < 10^−4^), and in patients who received tramadol with respect to those who did not receive either tramadol or tapentadol (2-sided significance *P* < 10^−4^ and *P* = 0.007, respectively).

**Graph 1:**
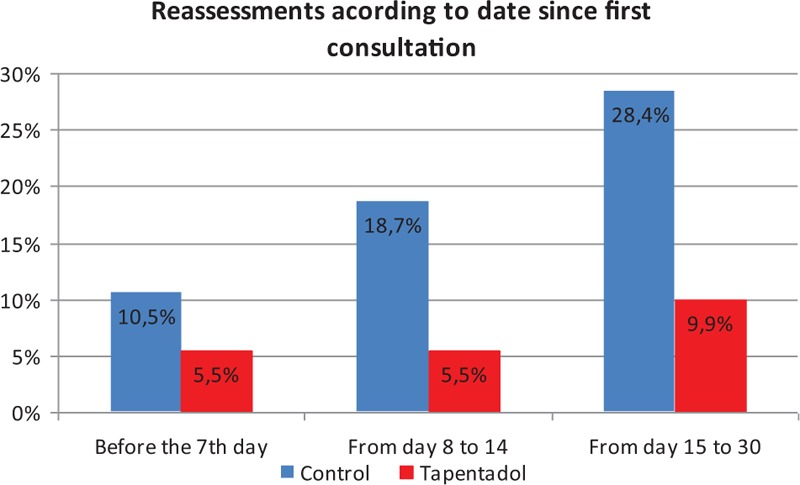
Reassessments according to date since first consultation.

Regarding the reassessments requested within the first month after the initial assessment according to the treatment administered, reassessments were less common in the tapentadol group compared with the control group (Graph 1), although in the first 7 days, the difference was still not significant (2-sided significance of *P* = 0.186). It was significant, however, at days 8 to 14 (*P* = 0.001, odds ratio [OR] 0.252 with 95% confidence interval [CI] 0.100–0.635) and days 15 to 30 (*P* < 10^−4^, OR 0.277 with 95% CI 0.136–0.563). In this respect, it was observed that patients who received tapentadol returned significantly later than those who did not receive either tramadol or tapentadol (2-sided significance *P* = 0.004) and especially compared with those to received tramadol (*P* < 10^−4^), who in turn also returned significantly earlier than those who received neither tramadol nor tapentadol (*P* < 10^−4^). No correlations were detected with the tapentadol or tramadol dose.

The time to the first reassessment requested tended to decrease very slightly for higher baseline VAS, with nonparametric correlation coefficients *ρ*_Spearman_ = −0.076 and *τ*_Kendall_ = −0.061, which, given the large number of cases (732) are significant with 2-sided significance *P* = 0.040 in both cases. No correlation was observed with the tapentadol dose administered.

**Graph 2:**
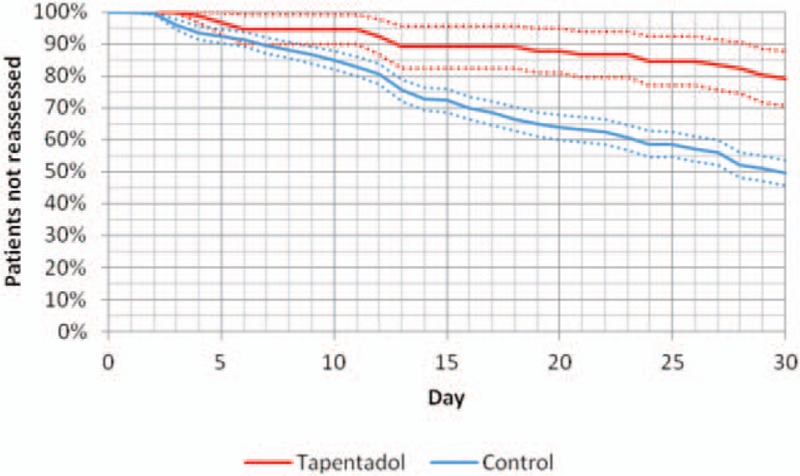
Proportion of patients in the tapentadol group and the control group who did not attend the ED for reassessment from the initial assessment and for the following 30 days (Kaplan-Meier curves). ED = emergency department.

Graph 2 shows the proportion of patients in the tapentadol group and the control group who did not attend the ED for reassessment from the initial assessment and for the following 30 days (Kaplan–Meier curves).

**Graph 3:**
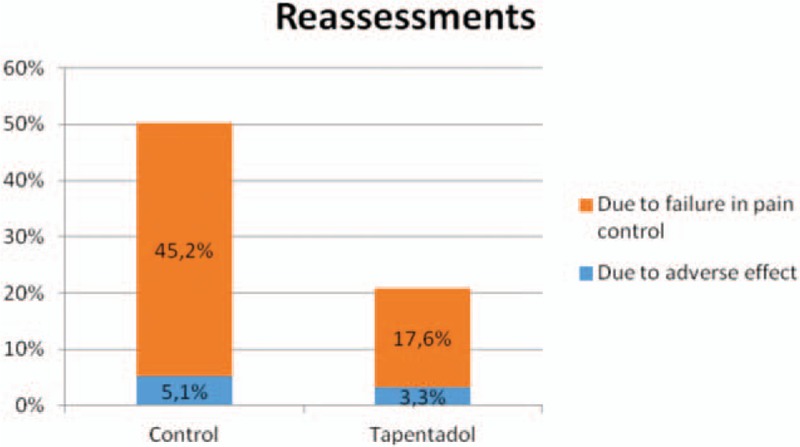
Percentage of reassessments in the first 30 days after the initial assessment in the ED. ED = emergency department.

In total, 50.3% of patients in the control group (323 patients, of whom 290 attended for pain and 33 for adverse effects) and 20.9% of patients in the tapentadol group (19 patients, of whom 16 attended for pain and 3 for adverse effects) visited the ED for reassessment in the first 30 days after the initial assessment in the ED, as shown in Graph 3. Of the first reassessments requested after the initial assessment, 89.5% were for pain (306 vs 36 for adverse effects). It is here in particular where there is a notable reduction in the tapentadol group compared with the control group (*P* < 10^−4^ in Fisher's exact test and OR 0.258 with 95% CI 0.147–0.453), whereas the reduction in the (few) reassessments requested for adverse effects is not significant.

## 4 Discussion

Pharmacological treatment for back pain can include paracetamol, metamizole, NSAIDs (conventional and cycloxigenase-2 inhibitors), minor opiates (tramadol), tapentadol and major opiates (buprenorphine, morphine, fentanyl and oxycodone), and depends on a range of factors including the pain intensity, cause of the pain and response to previous analgesic treatments.^[13,14]^

Tapentadol is a potent analgesic with a dual mechanism of action, as it contains μ-opioid receptor agonist activity and additional noradrenaline reuptake inhibition properties. It is indicated for the control of severe chronic pain in adults that can only be adequately treated with an opioid analgesic.^[12]^ Tapentadol treatment should be individualized, seeking the optimal dose that provides the patient with pain relief without the onset of toxic effects, and always under proper medical supervision. It is also available in an extended-release formulation (retard), which permits adequate pain control with twice daily administration.^[12]^ In clinical trials conducted on knee osteoarthritis and low back pain, tapentadol was superior to placebo and non-inferior to oxycodone in the reduction of pain intensity.^[15–19]^ Furthermore, in a systematic review that included 4 clinical trials—3 phase III clinical trials with 12-week follow-up and an open-label safety study with 52-week follow-up, in a total of 4094 patients with osteoarthritis, low back pain or both—extended-release tapentadol was associated with a reduction in pain intensity compared with placebo and oxycodone. However, the clinical significance of these results is limited, due largely to differences in the variables used to evaluate the efficacy in the reduction of pain intensity and the high withdrawal rates in the clinical trials analyzed.^[20,21]^

No studies have been published to date that have evaluated the impact of the different drug treatments available for the treatment of back pain on the demand for reassessments by patients due to poor pain control despite the treatment prescribed or due to the onset of treatment-induced adverse reactions.

In our study, it was observed that the group of patients initially treated with tapentadol demanded urgent reassessment within 30 days after the initial assessment less often than those who did not receive tapentadol, with this difference being more marked between 8 and 30 days after the initial assessment. This finding is consistent with the fact that patients treated initially with tapentadol had better clinical pain evolution (reduction in VAS score and an increase in SF-36 score) compared with those who did not receive tapentadol. In the set of patients analyzed, almost all the first reassessments requested were for poor pain control, with the proportion of patients significantly lower in those who received tapentadol compared with those who did not receive it.

It should be considered that, as this is a retrospective observational study, there were limitations in the availability of some clinical data, particularly in patients in the control group (baseline values for pain intensity). These limitations were taken into account when analyzing the data and interpreting the results. Other limitations related to the retrospective design of our study are the leak of assessment of the potential psychogenic component for ED visits that had been recently described and may be considered as a focus of interest in further research initiatives.

## 5 Conclusions

The prescription of adequate drug treatment for back pain in EDs—always after conducting a complete anamnesis and the pertinent examinations in each case—is essential to achieve optimal pain control, while at the same time avoiding the onset of adverse reactions and ultimately reducing the demand for reassessments in the ED by these patients.

In this study in patients with low back pain, tapentadol shows clear advantages as regards pain control and less need for reassessments in the ED over the other analgesics analyzed.
